# A novel entity of massive multifocal osteolyses in the elderly

**DOI:** 10.1016/j.bonr.2024.101765

**Published:** 2024-04-17

**Authors:** Patrick Orth, Phillip Rolf Stahl, Wolfgang Tränkenschuh, Daniel Baumhoer, Tim Kehl, Hans-Peter Lehnhof, Günther Schneider, Eckart Meese, Henning Madry, Ulrike Fischer

**Affiliations:** a Department of Orthopaedic Surgery, Saarland University Medical Center, D-66421 Homburg, Germany; b Center of Experimental Orthopaedics, Saarland University, D-66421 Homburg, Germany; c Department of Pathology, MSB Medical School Berlin, D-14197 Berlin, Germany; d Department of Pathology, Saarland University Medical Center, D-66421 Homburg, Germany; e Bone Tumor Reference Center, Institute of Pathology, University Hospital Basel, CH-4031 Basel, Switzerland; f Center for Bioinformatics, Saarland Informatics Campus, D-66123 Saarbrücken, Germany; g Department of Radiology, Saarland University Medical Center, D-66421 Homburg, Germany; h Institute of Human Genetics, Saarland University, D-66421 Homburg, Germany

**Keywords:** Osteolysis, Elderly, Novel entity

## Abstract

Osteolyses are common findings in elderly patients and most frequently represent malignant or locally aggressive bone tumors, infection, inflammatory and endocrine disorders, histiocytoses, and rare diseases such as Gorham-Stout syndrome. We here report on a novel entity of massive multifocal osteolyses in both shoulders, the right hip and left knee joint and the dens of an 83-year-old patient not relatable to any previously known etiopathology of bone disorders. The soft tissue mass is of myxoid stroma with an unspecific granulomatous inflammatory process, aggressively destroying extensive cortical and cancellous bone segments and encroaching on articulating bones in diarthrodial large joints. Radiological, nuclear medical, serological, histological, and immunohistochemical analyses were incapable of further classifying the disease pattern within the existing scheme of pathology. Quantitative polymerase chain reaction and next generation sequencing revealed that mutations are not suggestive of any known hereditary or acquired bone disease. Possible treatment options include radionuclide therapy for pain palliation and percutaneous radiation to arrest bone resorption while surgical treatment is inevitable for pathological fractures. This case study shall increase the awareness of the musculoskeletal community and motivate to collect further information on this rare but mutilating disorder.

## Highlights

• We report a novel entity aggressively devastating extensive bone segments.
• This disease cannot be classified within the existing nosological scheme.
• Treatment options include radiation and radionuclide therapy as well as surgery.
• This shall increase the awareness for a rare but mutilating disorder.

## 1 Introduction

**Fig. 1:**
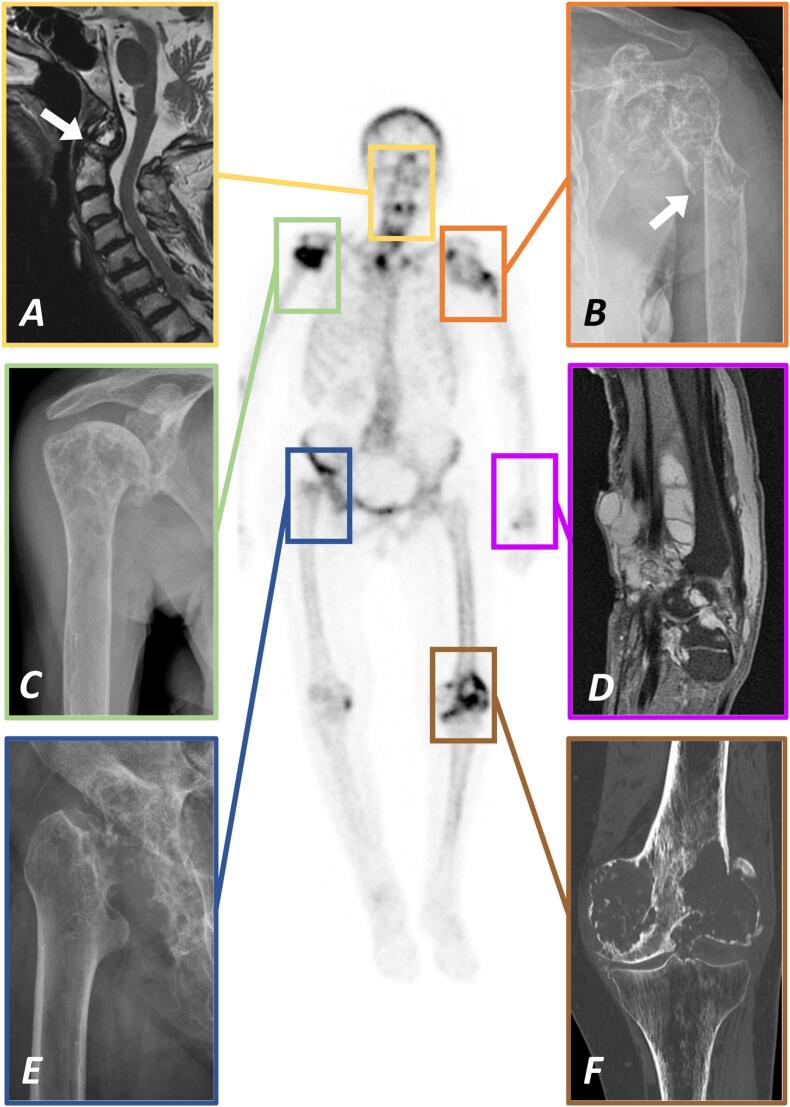
Bone scintigram showing the predominant skeletal manifestations of multifocal osteolyses in the 83-year-old female patient. Note the osteolytic destruction and undisplaced fracture of the dens (A; MRI T1 sagittal; white arrow pointing at the fracture site) which required fusion of C1 and C2 and the consolidated fracture of the left proximal humerus diaphysis following radiation therapy (B; X-ray anteroposterior; white arrow pointing at the fracture site) which was treated conservatively. The osteolysis of the right proximal humerus (C; X-ray anteroposterior) as well as the polycystic tumor surrounding the extensor tendons at the left wrist (D; MRI PD FS sagittal) did not receive any specific treatment. Radiation therapy was applied to the right hip joint (E; X-ray anteroposterior) as well as to the left distal femur and knee joint following distal femoral replacement due to a pathological fracture (F; CT coronal).

Osteolyses are common findings in elderly patients and most frequently represent metastatic bone lesions (Falkmer et al., 2003). Their differential diagnosis incorporates primary malignant or locally aggressive bone tumors, infection, inflammatory and endocrine disorders, histiocytoses, and rare diseases such as Gorham-Stout syndrome (Rosenthal, 1997). Among patients over 80 years of age, lytic bone lesions caused by these known entities are common findings. Despite the broad pathogenetic variety of osteolytic lesions, we here report and dissect an unusual case of an elderly female patient with massive destruction of extensive bone segments and large joints such as both shoulders, right hip and left knee as well as the dens (Fig. 1). To the best of our knowledge, such an entity has never been described before. We present our intensive and multidisciplinary quest for reaching a diagnosis in accordance with the existing literature and report on the clinical, imaging, and laboratory evidence as well as on histopathological and advanced molecular genetic analyses. Despite overarching diagnostic assessment, this severely disabling condition can neither be ascribed to any of the known etiologies nor classified within the existing pathology system. Yet, regarding the relevant clinical impact, we see the necessity to shed more light onto this rare disorder and increase its visibility among clinicians and researchers in the field of musculoskeletal medicine.

## 2 Methods and case presentation

A 83-year-old female patient first presented to our department in April 2016 with pain at the right groin and the left knee joint. Her past medical, family, and psychosocial history was noncontributory.

**Fig. 2:**
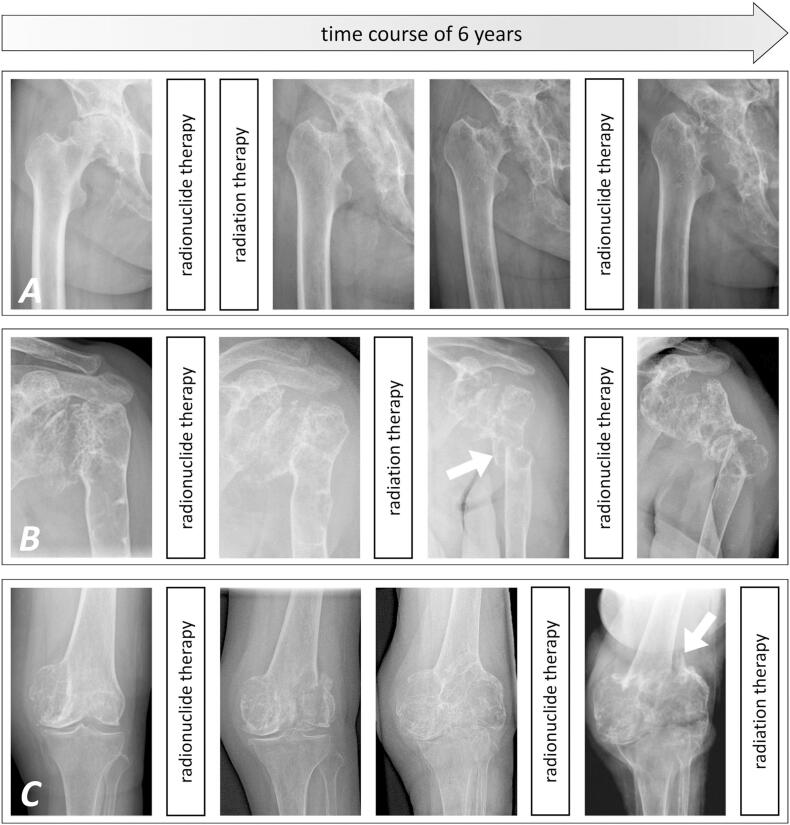
Anteroposterior X-rays of the right hip (A), the left shoulder (B) and the left knee joint (C) from similar time points demonstrating the rapidly progressive osteolytic destruction of the respective long bone segments and diarthrodial joints over the course of six years. These three locations have been selectively treated by radiation therapy (at the left knee following endoprosthetic replacement) while symptomatic off-label systemic radionuclide therapy by 153Sm-EDTMP was administered repeatedly and improved pain at all anatomic locations. White arrowheads in B and C point at the sites of pathological fractures of the left proximal humerus and distal femur diaphysis.

Bone scintigram revealed an uptake at the right hip joint, the left proximal humerus, and the left medial femoral condyle. X-rays and magnetic resonance imaging (MRI) investigations showed large osteolyses at the right supraacetabular ileum, the left glenohumeral joint, and the left medial femoral condyle (Fig. 2). Cancer staging by single photon emission computed tomography (SPECT) was unremarkable.

Blood samples revealed no signs for a rheumatic disease and detected no microbial pathogens. Despite a slightly elevated value for C-reactive protein (CRP) of 17.3 mg/l, leukocyte count (5.700/μl), rheumatoid factor (11 IU/ml), anti-citrullinated protein antibody (anti-CCP; <0.5 U/ml), anti-nuclear antibody screening (ANA; <1:80), human leukocyte antigen B27 (HLA-B27; negative), differential hemogram and electrophoresis were normal. Serum and urine analysis ruled out multiple myeloma. Soluble interleukin-2 receptor (sIL-2R) and tumor markers (carcinoma antigen (CA) 15–3, 19–9, 72–4, 125, TPA (tissue polypeptide antigen), NSE (neuron-specific enolase), SCC (squamous cell carcinoma antigen), AFP (alpha-fetoprotein), CEA (carcinoembryonic antigen), beta-2 microglobulin, S100) were not indicative of any specific oncological entity. Yet, parameters of bone metabolism were altered with elevated levels of alkaline phosphatase (232 U/l) and intact parathyroid hormone (307 pg/ml) *vis-à-vis* reduced (25-hydroxy) vitamin D3 (4.4 ng/ml). Serum levels were normal for calcium (2.5 mmol/l) and slightly decreased for phosphate (2.3 mg/dl) without evidence for renal or hepatic failure.

Incision biopsies of the left medial femoral condyle and the left shoulder showed fragments of lamellar bone without signs of malignancy and, in the background of a loose myxoid matrix, a sparse population of spindle-shaped cells with at the most slight pleomorphic nuclei and no staining reaction against p53, Mib-1 or AE1/3, accompanied by signs of a chronic inflammation. Histopathology detected no CD1a-positive cells and no signs for a lymphatic malformation. Microbiological analyses including fungal, bacterial, mycobacterial, and atypical organism culture, specific stains, and polymerase chain reaction ruled out an infectious cause of the osteolyses.

Because of increasing pain at the right hip, bone scintigram revealing a pronounced uptake, and conventional joint replacement surgery being unfeasible -due to expeditious progression of local bone destruction-, an off-label radionuclide therapy (Fig. 2) was initiated in September 2016. Intravenous administration of samarium 153-ethylene diamine tetramethylene phosphonate (153Sm-EDTMP) was well tolerated and relieved the patient's symptoms, enabling her to walk distances up to 500 m.

Yet, computed tomography (CT) and MRI of the right hip and hemipelvis showed progressive osteolyses in March 2017. Our interdisciplinary tumor board consented to an additional percutaneous radiation therapy to impede disease progression. Applying a cumulative dose of 40 Gray (Gy) in single fractions of 2 Gy in April 2017, the radiation resulted in subtle recalcification although not yielding further pain relief.

In November 2017, severe pain in the left shoulder was caused by progressive destruction of the proximal humerus and glenoid. As surgical options were limited and scintigraphic uptake was only moderate, we performed percutaneous radiation therapy (40 Gy). An undisplaced fracture of the proximal humerus diaphysis occurred in July 2018 and was treated conservatively by a cast and splint (Fig. 2).

**Fig. 3:**
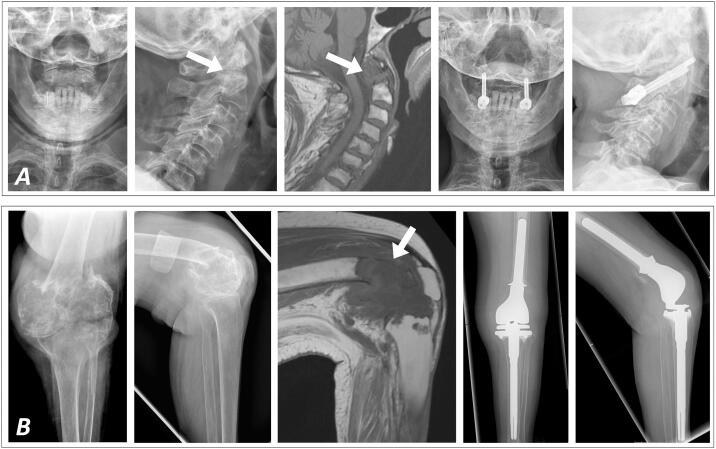
Both the dens (A; pre- and postoperative X-rays anteroposterior and lateral as well as MRI T1 sagittal) and the left distal femur diaphysis (B; pre- and postoperative X-rays anteroposterior and lateral as well as MRI T1 sagittal) sustained pathological fractures requiring operative treatment. As osseous destruction at both sites was advanced, osteosynthesis was not feasible anymore but fusion of C1 and C2 was necessary at the cervical spine while distal femur resection and reconstruction by tumor endoprosthetic replacement was performed at the left femur and knee joint, followed by radiation therapy. The postoperative course at both locations was uneventful, the patient was mobilized without restrictions and free of complaints after the rehabilitation.

Two months later, the patient suffered from acute pain in the neck. X-rays and MRI showed an undisplaced fracture of the dens due to massive osseous destruction (Fig. 3), requiring fusion of C1 and C2 by screws.

Due to progressive pain at the left shoulder from March 2019 on -despite stable fracture consolidation-, a systemic off-label radionuclide therapy (153Sm-EDTMP) was performed again in May 2019, providing considerable pain relief.

In May 2020, a painless soft tissue mass developed at the dorsal aspect of her left wrist (Fig. 1). MRI depicted a large polycystic tumor surrounding the extensor tendons, radiologically classified as ganglion cysts and not requiring further treatment.

The patient sustained a displaced pathological fracture of the left distal femur diaphysis in September 2020 (Fig. 3). Distal femur resection and endoprosthetic replacement (GMRS/MRH, Stryker, Kalamazoo, MI, USA) was followed by percutaneous radiation (40 Gy). During radiation therapy, the patient suffered from a refracture of her left proximal humerus diaphysis, which was treated conservatively and resulted in bone consolidation with acceptable angulation malalignment. Currently, the patient is in a stable condition regarding the musculoskeletal system and mobilized with the aid of a walker and wheelchair. On demand pain therapy with non-steroidal anti-inflammatory drugs is sufficient in everyday life. No new painful or deformed skeletal sections have appeared in the meantime.

**Fig. 4:**
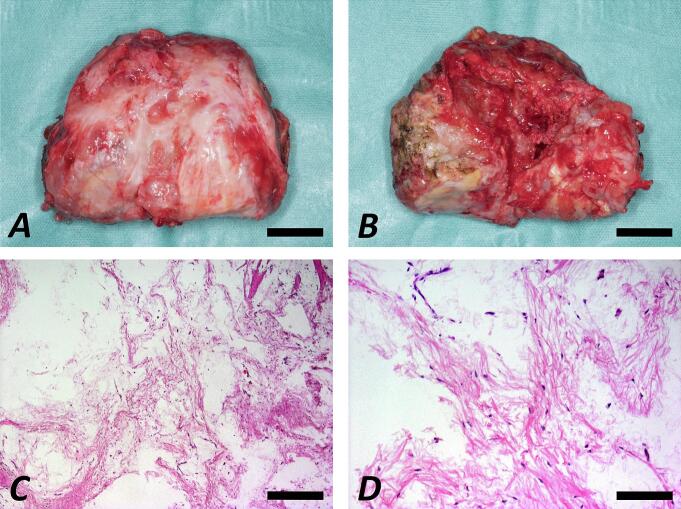
Macroscopic images depicting the left distal femur following resection and distal femoral replacement in the course of a pathological distal femur fracture (A: view from anterior; B: view from posterior; scale bars 20 mm). Histological examination following hematoxylin-eosin staining showing fragments of lamellar bone and a sparse population of spindle-shaped cells with only slight pleomorphic nuclei, in the background of a loose myxoid matrix, accompanied by few thin-walled vessels (C: magnification 5×, scale bar 400 μm; D: magnification 20×, scale bar 100 μm).

Histopathological analysis of the left distal femur (Fig. 4) showed loose myxoid stroma with few CD68-posititive but S100-, p53-, and AE1/3-negative cells. Signs of malignancy, hemangiomatosis/lymphangiomatosis, and emperipolesis were not present. Molecular pathological evaluation excluded FUS-CHOP fusion transcripts and simultaneous CDK4- and MDM2-amplification. Mutation analysis showed wild type of the GNAS gene.

**Fig. 5:**
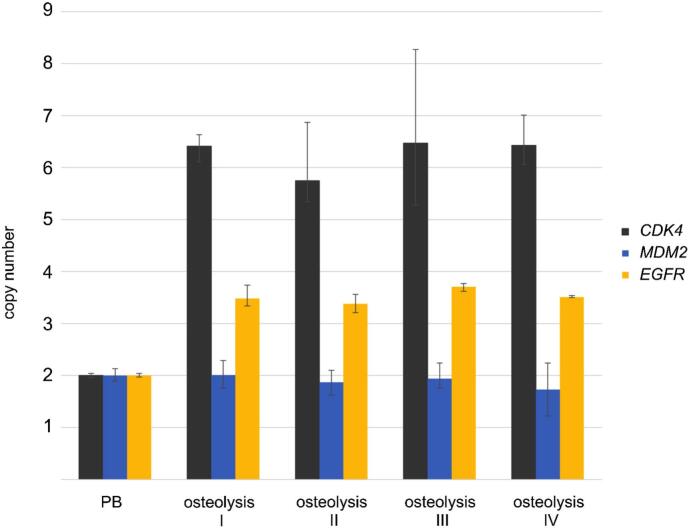
Copy number analysis of CDK4, MDM2 and EGFR using quantitative polymerase chain reaction (qPCR). Copy numbers were analyzed by qPCR using TaqMan copy number assays. RNaseP was used as reference gene in the TaqMan assays and DNA from normal blood lymphocytes from the patient (PB) served as standard for normal diploid copy number. Copy numbers are shown as mean from four technical replicates with vertical lines indicating the range. Osteolysis samples I, II, III and IV revealed CDK4- and EGFR-amplification but no simultaneous MDM2-amplification.

Comprehensive molecular genetic analyses investigated copy number/gene amplification using quantitative PCR (qPCR) and TaqMan probes for the CDK4, MDM2 (chromosome 12q13–15), and EGFR (chromosome 7p11.2) genes as well as next generation sequencing (NGS) for mutation analysis. In brief, DNA was isolated using the chloroform/NaCl method and the patient's blood lymphocytes (PB) were used as control standard for normal diploid copy number. The MGIEasy DNA Library Prep Kit (PE100) was applied for whole genome sequencing. Data preprocessing was performed using the genome analysis toolkit (GATK) and variant calling using Mutect2. We detected CDK4 and EGFR amplification in four DNA extractions from different areas of the left distal femur but failed to detect MDM2 amplifications (Fig. 5). NGS excluded mutation of the GNAS gene while multiple other genes revealed mutations (Supplementary Information). Regarding the synopsis of clinical, radiological or histopathological findings, none of these mutations cause bone disorders comparable to the here presented disease.

## 3 Discussion

According to broad and sophisticated diagnostic analyses, the here presented destruction of extensive skeletal segments cannot be assigned to any previously known entity of bone or soft tissue malformation and thus represents a novel entity in the field of musculoskeletal medicine.

Several differential diagnoses were considered during the course of treatment (Table 1). The disseminated decay of multiple large bones and joints renders the presence of a primary bone sarcoma very unlikely and biopsies excluded malignancy. Osseous metastases rather than primary bone sarcoma tend to affect multiple skeletal regions, in theory being more in accord with the radiological finding of multiple osteolyses. Yet, histopathological and immunohistochemical results did not allude to the presence of bone metastases and staging investigations were negative. Oncological disorders originating from the bone marrow such as multiple myeloma or multiple solitary plasmacytomas (Ozbayrak et al., 2013) were ruled out by histological and serological analyses.

**Table 1:** Potential differential diagnoses and distinguishing features of the novel osteolytic entity.

Differential diagnosis	Distinguishing feature of novel entity
Primary bone sarcoma	Malignancy excluded by histology No history of cancer
Bone metastases	Malignancy excluded by histology No history of cancer No primary cancer upon staging
Osteomyelitis/empyema	No microbial pathogens detected in situ No bacteremia No clinical/serological signs of infection
Rheumatoid arthritis	Negativity for RF, anti-CCP, ANA, HLA-B27 in serum and tissue samples Unremarkable hemogram and electrophoresis No clinical signs of rheumatoid arthritis
Multiple myeloma/ Multiple solitary plasmacytoma	No evidence by histology, serology, and electrophoresis
Giant cell tumor	No giant cells/osteoclasts upon histology
Cyst-like brown tumors	No osteoclast formation and hemosiderin deposition upon histology
Intraosseous lipoma/ Atypical lipomatous tumor	No lipoblasts upon histology Lack of simultaneous CDK4-/MDM2-amplification
Skeletal myxoma	Epiphyseal involvement with joint destruction Multifocal osseous involvement Lack of GNAS mutation
Constitutional bone disorders	Negative family history of bone disorders Late occurrence (> 80 years) Lack of accordant molecular genetic mutation analysis
Gorham-Stout syndrome	No lymphangiomatosis /hemangiomatosis upon histology Multifocal osseos involvement (not affecting the cranium) Late occurrence (>80 years) Fracture consolidation
Langerhans cell histocytosis	Negativity for CD1a upon immunohistochemistry
Rosai-Dorfman disease	No emperipolesis upon histology Negativity for S100 upon immunohistochemistry
MONA/Torg/Winchester syndrome	Lack of accordant molecular genetic mutation analysis
Erdheim-Chester disease	No sclerotic but osteolytic lesions on X-ray and CT Lack of BRAF^V600E^ mutation No perirenal infiltration/periaortic sheathing No xanthelasma/exophthalmos

Benign but locally aggressive bone tumors such as giant cell tumors and intraosseous lipoma may also result in massive osteolyses. However, neither giant cells nor lipoblasts were detected by histology and the lack of simultaneous CDK4- plus MDM2-amplification ruled out an atypical lipomatous tumor/well-differentiated liposarcoma. Skeletal myxoma usually do not result in joint destruction, do not affect the epiphyses, and are of unicentric origin (Stout, 1948). Further, the GNAS mutation analysis was negative, making skeletal myxoma an improbable diagnosis.

The International Skeletal Dysplasia Society publishes the Nosology and Classification of Genetic Skeletal Disorders (Mortier et al., 2019). The vast majority of these 461 constitutional diseases, e.g. idiopathic multicentric osteolyses (Goldfarb et al., 2012), arise almost exclusively during infancy and hardly ever in the elderly. Specifically, homozygous inactivating mutations of the matrix metalloproteinase 2 (MMP2), MMP14, or SH3PXD2B genes may cause defective collagen remodeling (de Vos et al., 2019) and result in the allelic variants (Zankl et al., 2007) of MONA (multicentric osteolysis, nodulosis and arthropathy) (Bhavani et al., 2021), Torg (Zankl et al., 2007) or Winchester (Evans et al., 2012) syndrome. These entities were however ruled out by molecular genetic analysis.

Gorham-Stout syndrome (GSS) was first reported in 1955 (Gorham and Stout, 1955) and presents non-hereditarily with a large osteolysis not respecting joint limits. This osteolysis in GSS usually affects one single portion of the skeleton, most frequently the cranium and shoulder or pelvic girdle (El-Kouba et al., 2015), albeit multicentric affections have been described (Vigorita et al., 2006). The syndrome is most commonly recognized in children and young adults with an average age at diagnosis of 14 years for primary and 30 years for secondary cases following trauma (Tanoue et al., 2018). In GSS, pathological fractures rarely consolidate (Gowin and Rahmanzadeh, 1985). All of these typical characteristics differ from our case. Most importantly, the essential histological finding in GSS of lymphangiectatic tissue destroying bone matrix (Vigorita et al., 2006; Choma et al., 1987) was lacking here.

Rosai-Dorfman disease (RDD) is a very rare variant of histocytosis characterized by abundant histocytes causing cervical lymphadenopathy. According to the Histiocyte Society (Emile et al., 2016), extranodal RDD may also affect bone tissue and -very rarely- multifocal osseous involvement is the sole manifestation of the disease (Sundaram et al., 2005). Histological sections of RDD typically demonstrate emperipolesis by histiocytes; a finding which is pathognomonic for the disease (Emile et al., 2016). Upon immunohistochemistry, histiocytes are positive for S100, CD68 and CD163 but negative for CD1a. In contrast, the most important differential diagnosis for RDD is Langerhans cell histocytosis (LCH) which can be distinguished by positivity for CD1a and absence of emperipolesis. Here, tissue samples stained negative for CD1a, ruling out LHC. Moreover, emperipolesis was not detected and immunoreactivity to S100 was negative. Thus, we found no evidence for histiocytoses causing the osteolyses; specifically, RDD and LCH were reliably eliminated.

Empyema and osteomyelitis are further etiopathologies of osseous arrosion and joint destruction. Skeletal tuberculosis has been reported to cause multifocal bone and joint destruction simulating sarcoma (Fernández-Ruiz et al., 2016). Yet, these diagnoses require bacteremia or even sepsis in the past medical history and would have been confirmed by detection of microbial pathogens. Aseptic inflammatory joint diseases, i.e. rheumatic disorders such as rheumatoid arthritis, may also lead to mutilating joint destruction if left untreated. Neither in the blood samples nor within the tissue samples signs for a rheumatic disease were present.

Finally, alterations in the parameters of bone metabolism pointed at a late stage secondary or early stage tertiary hyperparathyroidism ascribed to alimentary vitamin D deficiency and was addressed by substitution. Importantly, a long-lasting hyperparathyroidism may result in osteitis fibrosa cystica and formation of cyst-like brown tumors (Rodríguez-Gutiérrez and Hinojosa-Amaya, 2015), but the histological findings revealed neither increased osteoclast formation/activity nor hemosiderin deposition. Besides, elevated serum alkaline phosphatase activity ruled out adult hypophosphatasia (Fenn et al., 2021).

Taken together, we have excluded all common and rare causes for multifocal large osteolyses in this elderly patient (Table 1). As no comparable condition is described to date, treatment options are not standardized and must resort to existing therapeutic modalities, demanding a trial-and-error approach. Off-label radionuclide therapy applying 153Sm-EDTMP markedly improved pain, but was incapable of arresting bone resorption. Vice versa, subtle recalcification was achieved by additional percutaneous radiation therapy, which in turn did not achieve pain palliation and possesses a potential risk of carcinogenesis. By combining both treatment modalities, we were able to relieve the patient's symptoms and delay disease progression for six years up until now with surgical treatment having been unavoidable only for the pathological fractures of dens and distal femur.

In a variety of osteolytic disorders, for example MONA syndrome (Pichler et al., 2016), bisphosphonates may relieve skeletal pain. It remains to be elucidated whereas experimental therapeutic strategies applying antiresorptive (bisphosphonates) or osteoanabolic drugs (e.g. teriparatid), alone or in combination with other agents such as interferon alpha-2 A (Löhr et al., 1995) or alpha-2B (Lopez-Gutierrez et al., 2012), may beneficially affect the treatment of this novel entity in the future. In this context -and to avoid potential side effects of radiation and radionuclide therapy- the inhibition of RANKL (receptor activator of nuclear factor kappa Β ligand) by denosumab represents an interesting novel therapeutic option and will have to be considered in case of disease progression.

Limitations of this case study such as the uncertainty regarding optimal diagnostic and therapeutic approaches or the necessity for experimental treatment measures are due to the lack of similar case descriptions. Strengths include the broad and interdisciplinary range of robust diagnostic modalities applied to compare this entity with known hereditary or acquired bone lesions. This peculiar case shall increase the awareness of the musculoskeletal community for a rare but severely mutilating disorder and stimulate further research to identify its cause and define a specific therapy.

## Abbreviations

RF: rheumatoid factor
CCP: citrullinated protein antibody
ANA: anti-nuclear antibody
HLA: human leukocyte antigen
CD: cluster of differentiation
MDM: mouse double minute
GNAS: guanine nucleotide binding protein, alpha stimulating activity polypeptide
MONA: multicentric osteolysis, nodulosis and arthropathy
CT: computed tomography

## Funding

This research did not receive any specific grant from funding agencies in the public, commercial, or not-for-profit sectors.

## CRediT authorship contribution statement

**Patrick Orth:** Writing – review & editing, Writing – original draft, Visualization, Validation, Supervision, Project administration, Methodology, Investigation, Formal analysis, Data curation, Conceptualization. **Phillip Rolf Stahl:** Writing – review & editing, Writing – original draft, Validation, Methodology, Investigation, Formal analysis, Conceptualization. **Wolfgang Tränkenschuh:** Writing – original draft, Validation, Supervision, Methodology, Investigation, Formal analysis, Data curation. **Daniel Baumhoer:** Writing – review & editing, Validation, Resources, Methodology, Investigation, Formal analysis, Data curation. **Tim Kehl:** Writing – review & editing, Validation, Software, Methodology, Investigation, Data curation, Conceptualization. **Hans-Peter Lehnhof:** Writing – review & editing, Validation, Software, Methodology, Investigation, Formal analysis, Conceptualization. **Günther Schneider:** Writing – review & editing, Supervision, Resources, Methodology, Investigation, Formal analysis, Conceptualization. **Eckart Meese:** Writing – review & editing, Supervision, Software, Resources, Methodology, Investigation, Data curation, Conceptualization. **Henning Madry:** Writing – review & editing, Visualization, Validation, Supervision, Resources, Methodology, Investigation, Formal analysis, Data curation, Conceptualization. **Ulrike Fischer:** Writing – review & editing, Visualization, Validation, Supervision, Resources, Methodology, Investigation, Formal analysis, Data curation, Conceptualization.

## Declaration of competing interest

Patrick Orth, Phillip Rolf Stahl, Wolfgang Tränkenschuh, Daniel Baumhoer, Tim Kehl, Hans-Peter Lehnhof, Günther Schneider, Eckart Meese, Henning Madry, and Ulrike Fischer declare that they have no conflict of interest. This article does not contain any studies with human or animal subjects. Informed consent was obtained from the patient for being included in the study.

## Data Availability

Data will be made available on request.

## Appendix A Supplementary data

Supplementary material Image 1
